# Progress in Diseases of the Blood

**Published:** 1901-12-14

**Authors:** 


### Progress in Diseases of the Blood.

**Lymphadenoma.**—J. Michell Clarke[1] discusses the difficult question, Is there a single specific disease to which we may give this name or the more or less synonymous terms Hodgkin's disease, adénie, malignant lymphoma or pseudoleukemia ? He concludes that there is such a disease, infective in character, and acute or chronic in type, but that it must be distinguished from tuberculous affections, lymphosarcoma and lymphatic leukemia. It causes a generalised affection of the lymph glands, usually commencing in the cervical ones, most frequent in males in the first half of life, showing at first few changes in the blood, though as it advances there is a diminution of red cells and Hb., and sometimes an increase of the polynuclear leucocytes. Various organisms have been found in the blood, and direct infection has been seen, but it is uncertain what the special organism is. Cases due to tuberculosis may show very similar symptoms. The term lymphosarcoma is better confined to cases where one or more contiguous groups of glands are affected, the capsules are perforated, and the surrounding tissues invaded, metastases being produced without generalised gland invasion. Acute leukemia may be difficult to distinguish except that the large or small lymphocytes are usually in excess. Chronic lymphatic leukemia shows also a larger spleen and the typical blood changes, while a leucocytosis in lymphadenoma affects chiefly polynuclear cells. Other diseases having a superficial resemblance are splenic anæmia, the pseudo leukemia of children, status lymphaticus, and persistence of the thyroid. G. L. Gulland[2] is inclined to think that lymphadenoma is really a form of tuberculosis, though observers have failed in some cases in getting any evidence of the fact. Indeed, a differential count of the leucocytes and bacterial examination of the glands is most important if we are to come to any definite conclusion on this disease.

**Splenic Anæmia.**—H. Maxon King[3] reports the case of a woman, aged 58, who had been of a pale yellow colour for some years, and suffered from diarrhœa and melæna for 18 months. The spleen filled one side of the abdomen. The red cells only numbered 875,000, Hb. 15, white cells 3,150, megaloblasts 6·5 per cent., and there was marked poikilocytosis. There were 4 per cent. of myelocytes, and the other white cells were nearly normal. The writer has never seen such marked blood changes except in pernicious anæmia. N. E. Brill[4] corroborates the account given by Bovaird of *primary splenomegaly* and adds three cases which occurred in one family. The condition lasted ten or fifteen years, and two patients are still in fair health ; anæmia was absent for ten years and not marked then, the spleen was enormously enlarged and the liver moderately. There were occasional hæmorrhages, profuse sweating, a brownish-yellow colour of the skin, and a yellow conjunctival thickening in each eye, but no stunting of the growth, clubbed fingers, or ascites.

**Histology of the Blood.**—Engel[5] holds that the red cells are derived from the nucleated red cells of the marrow. These are a highly specialised and late development, only present in mammals, by which the cell loses its power of reproduction and becomes simply a carrier of oxygen. In serious diseases the change may be imperfectly carried out. Thus, where there is a great increase of nucleated red cells in the marrow there is a deficiency of normal cells and Hb. in the blood, as in chlorosis. Sometimes this may go on to the appearance of nucleated red cells. If the yellow marrow be changed to red, megaloblasts are found in the blood, as in pernicious anæmia. When the red marrow loses its nucleated red cells and its leucocytes, marked anæmia and the disappearance of all forms of leucocytes, except lymphocytes, ensues. H. Vaquez[6] suggests that the resisting power of red cells, when placed in unfavourable salt solution, may be of importance in the study of disease. As solutions of less and less strength are employed one is found in which the cells are seen to swell up and lose their Hb. and the blood becomes gelatinous. The point at which these changes begin for human blood seems to be a solution of ·44 to ·48 per cent. In jaundice and malignant disease the resisting power of the red cells is increased. In a research as to the hæmoglobin contents of the red blood corpuscle G. H. Gouldsmith[7] finds the Hb. value of the cell in the blood of women is 89·7 per cent. of that of men, and the average corpuscle in women is smaller than that in men as 8 is to 11. To facilitate the estimation of the hæmoglobin he recommends the use of an instrument containing a standard solution of blood shaken up with coal gas, which is permanent. The specimen to be examined is diluted and shaken up with the same gas, so that we are comparing two solutions of C.O. hæmoglobin. Ehrlich[8] has invented an eyepiece with a square diaphragm for counting red and white cells in the same preparation. Thus the white cells can be counted and the diaphragm reduced so that the field is one-ninth or one-sixteenth of the previous one and it then becomes easy to count the red corpuscles. J. G. Emanuel[9] has a useful discussion of the clinical side of blood changes. He classifies the cells as :—

I. *Red* including (A) Erythrocytes, or normal red cells, sometimes varying in size.

(B) Normoblasts, or nucleated immature red cells, showing active regeneration in any severe anæmia.

(C) Megaloblasts, a degenerative embryonic form in fatal cases, showing the failure of repair.

II. *White granular* ones, derived from bone marrow, including :

(D) Polymorphonuclear neutrophiles forming 70 per cent. of normal white cells.

(E) Polymorphonuclear eosinophiles forming 3 per cent. of normal white cells.

(F) Polymorphonuclear basophiles (mast cells) forming ·5 per cent. of normal white cells.

(G) Mononuclear neutrophilus or myelocytes.

(H) Mononuclear eosinophiles or myelocytes.

These two last are only seen in the blood during disease, but all five varieties may be found at any time in the marrow. Besides these, we have :—

III. *Non-granular* forms derived from the lymph glands, as :

(I) Small lymphocytes forming 23 per cent.

(J) Large lymphocytes forming 4 per cent.

(K) Transitional forms or varieties of the last class.

Now in those secondary anæmias which result from hæmorrhage, even though it be a small one, there is leucocytosis which distinguishes, *e.g.*, a cerebral hæmorrhage from concussion. In the severest forms of secondary anæmias we may get megaloblasts, but they are never in excess of the normoblasts, while in pernicious anæmia they are. In the latter we have, too, the great reduction of red cells, many of which are deformed ; there are myelocytes, and an excess of lymphocytes. In myelogenous leukemia the marrow is affected, and hence we find an excess of normoblasts and some megaloblasts. There is also a great number of myelocytes (35 per cent.), with some excess of lymphocytes, eosinophiles, and mast cells. In the lymphatic form, on the other hand, the nucleated red cells are rare, but the lymphocytes are 95 per cent., while the polynuclear sink to 4 per cent., with perhaps 1 per cent. of myelocytes. In rapidly growing malignant disease we find leucocytosis and some myelocytes. An excess of eosin cells is seen in infants, in some skin diseases, in leukemia, in true bronchial asthma about the time of a paroxysm, in those suffering from intestinal worms, after pneumonia, acute rheumatism, and after other fevers, or in tumours affecting bones and in chronic tumours of the spleen. The *influence of Ether* anæsthesia on the blood has been the subject of considerable discussion. J. B. Blake [10] denies that it has any effect apart from that of the operation itself, while Da Costa thinks that precautions, such as previous purgation of the subjects of inquiry, are necessary, and comes to the conclusion that there is an increase of the red cells, and fall of Hb. when ether is used. B. S. Curtis believes that there is a great increase of leucocytes. The question seems to be by no means settled, any more than the amount of risk incurred in operations where great anæmia is present. The freezing point of blood has been put forward as a test for various diseases, but in the case of typhoid Rumpel [11] finds that the asserted alteration from the normal does not exist, the freezing-point in his cases at different stages of the disease being nearly normal.

**The Blood in Scarlatina.**—F. P. Mackie [12] records the examination of 25 cases and remarks on the frequency of moderate anæmia and a reduction of Hb. to an equal degree. Leucocytosis begins 24 hours later than the rash, reaching a maximum in mild cases 24 hours after its disappearance. The chief increase seems to be in polynuclear cells with some increase of eosinophiles. A deficiency of leucocytosis in a severe case is a bad sign, and a high rate often indicates some deep suppuration. He denies Cabot's statement that the leucocytosis may appear even before the rash, and notices the value of a blood examination in the diagnosis from measles in which there is no leucocytosis at all. The increase in eosinophiles may be related to the skin affection, for it is common to several skin diseases.

**Gelatine as a Hæmostatic.**—Horatio C. Wood [13] remarks that solutions of gelatine solidify on cooling if stronger than 2 per cent., but strong solutions of the iodides or chlorides prevent this change. When injected into an animal coagulation is much quickened, and true clotting, not mere jellification, takes place. Gelatine can oppose the action of peptone, which prevents clotting, and the statement that being non-dialysable it cannot be absorbed from the tissues is without foundation, because, first, it could be carried by the lymphatics, and, secondly, it is actually found to be absorbed, and the blood of the animal so treated clots in a fraction of the time required before injection. It has been used with success in aneurysms, purpura, hæmoptysis, and after operations. The question whether it can be administered by the mouth without destruction through digestion is disputed, but in some cases severe hæmorrhages have ceased after gelatine has been swallowed. Externally it has been used with success in epistaxis, etc., in a strength of 10 per cent. For hypodermic use a 2 per cent. solution (in normal salt solution) is sterilised by being raised to the boiling point at intervals for a short time. About $3\frac{1}{2}$ ounces of this, or 100cc. containing 30 grains of gelatine, are injected into the loose subcutaneous tissue with due precautions. Some fear that it might cause intravascular clotting has been expressed, but the writer thinks that the danger from this source is small. Moyniham [14] has found the injections useful in checking hæmorrhage in cancer, hæmophilia, and in operation on the kidney, or for chronic obstruction of the gall bladder. Injections are made at intervals of two to nine days, and seem to cause some pain, as well as a rise of temperature to 100°, which does not become normal for 48 hours. He generally uses six ounces of a 2 per cent. solution well sterilised. Some cases, however, have occurred of so-called tetanus following these injections with fatal results. It has been argued by E. W. Hey Groves [15] that this was probably not due to any infection but to thrombi from the increased coagulability, the convulsions being analogous to those in puerperal eclampsia, often called uræmic.

[1] Brit. Med. Jour., Sept. 14. [2] Ibidem. [3] Med. News, March 23. [4] Amer. Jour. Med. Sc., April. [5] Med. Chron., March. [6] Treatment, March. [7] Brit. Med. Jour., Sept. 21. [8] Glasgow Med. Jour., May. [9] Birmingham Med. Rev., June. [10] Med. Record, May 18. [11] Amer. Jour. Med. Sc., May. [12] Lancet, Aug. 31. [13] Merck's Archives, Aug. [14] Brit. Med. Jour., Sept. 14. [15] Ibidem

